# A responsive Co(ii) ^19^F PARAShift probe: activation of Fermi contact interactions triggered by pH-dependent coordination changes

**DOI:** 10.1039/d6cc00957c

**Published:** 2026-03-09

**Authors:** Kathleen M. Scott, Rahul T. Kadakia, Christopher D. Hastings, Georgia E. F. Barone, Jackson A. Reyna, Jin Xiong, Yisong Guo, Emily L. Que

**Affiliations:** a Department of Chemistry, The University of Texas at Austin Austin Texas 78712-1224 USA emilyque@cm.utexas.edu; b Department of Chemistry, Carnegie Mellon University Pittsburgh PA 15213 USA jinxiong@andrew.cmu.edu

## Abstract

We report a novel ^19^F MR imaging probe CoNO2ASF_5_ exhibiting a large ^19^F chemical shift change of over 30 ppm in response to physiological pH changes. Calculations identify Fermi contact interactions drastic as the mechanism of enhanced ^19^F MR shift.

## Abstract 2

We report a novel ^19^F MR imaging probe CoNO2ASF_5_ exhibiting a large ^19^F chemical shift change of over 30 ppm in response to physiologically relevant pH changes.

## Graphical abstract

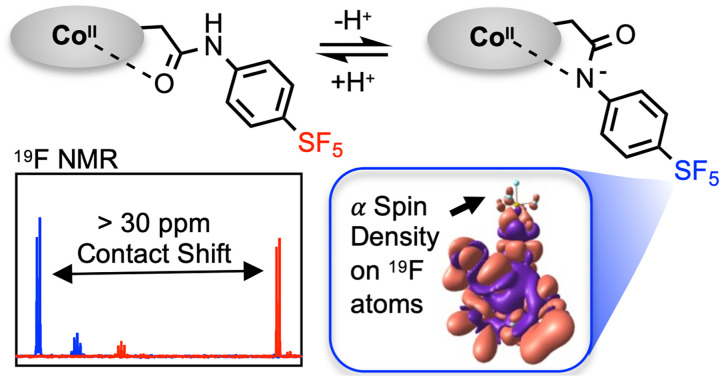

Mammalian blood acts as a buffer to maintain pH homeostasis at 7.4 and aberrations from this pH are associated with several pathologies including metabolic and respiratory acidosis/alkalosis, chronic obstructive pulmonary disease, and pneumonia.^1^ In cancer, tumor microenvironments are often acidic, primarily due to increased anaerobic glycolysis causing higher concentrations of lactic acid.^2–4^ Thus, accurate pH mapping has the potential to be a powerful diagnostic tool. While sensors exist to monitor biological pH (colorimetric, optical imaging, positron emission tomography), magnetic resonance imaging (MRI) has the distinct benefit of being nonionizing with infinite penetration depth, making this a promising modality for developing non-invasive, *in vivo* quantitative pH sensors.^5–10^

MRI is a widely used clinical imaging technique characterized by high spatial resolution and contrast. Traditional MRI detects ^1^H; however, due to the high proton concentration in living organisms, ^1^H MRI contains unavoidably high background signal which lowers specificity and quantitative power. As an alternative nucleus, fluorine (^19^F) has favorable properties: 100% ^19^F natural isotopic abundance, a nuclear spin of ½, excellent receptivity (83% of proton), and a large chemical shift range (>350 ppm). Given the lack of MR active fluorine in the body, quantitative images of biological environments can be taken with higher specificity than ^1^H MRI.

Paramagnetic metals can modulate chemical shifts in ^19^F MR imaging agents *via* pseudocontact shift (PCS) and Fermi contact (FC) interactions. PCS derives from through-space magnetic dipole interactions in which a frequency shift is caused by the anisotropy of the paramagnetic species’ magnetic susceptibility. ^19^F PARAshift agents commonly employ PCS as the metal fluorine distance is often in the 5–10 Å range.^11–14^ Cobalt(ii) has a relatively large PCS range (26 Å) due to fast Orbach relaxations causing a short electronic relaxation time (∼10^−12^ s).^15^ We have reported redox responsive ^19^F PARAshift probes that use a spin change from paramagnetic (PCS-active) Co(ii) to diamagnetic Co(iii) using an amide-linked perfluoro-*tert*-butyl ^19^F tag.^13,16,17^ However, with PCS-based probes the shift range has been limited (∼3–10 ppm). Cobalt(ii) probes have been developed for ^1^H PARAshift and Chemical Exchange Saturation Transfer (CEST) that utilize PCS and FC to induce proton frequency shifts.^18–21^ FC interactions result from direct overlap of unpaired electron spin on the MR active nucleus and can result in much larger chemical shift changes compared to PCS.^22,23^ However, effects are generally limited to a couple bond lengths away from the paramagnetic center and have not been widely exploited in responsive ^19^F MR sensors.

We report CoNO2ASF_5_, a responsive ^19^F MR probe that exhibits a >30 ppm ^19^F chemical shift change between acidic and basic environments. This probe contains an anilide-linked pentafluorosulfanyl moiety (–SF_5_) as our ^19^F reporter. This SF_5_ group's ^19^F signal (∼+60 ppm) is highly shifted from CF_3_ groups (∼−70 ppm), and thus these tags could be used in tandem to monitor multiple analytes simultaneously. Further, despite inequivalent fluorines splitting the SF_5_ signal, studies successfully employ this tag as a sensitive, biocompatible ^19^F reporter group.^24,25^ Crucially, using an anilide moiety, as opposed to an alkyl amide, imbued new coordination properties to enable pH-responsiveness within a physiologically relevant range and activation of FC interactions several bonds away from the Co(ii) center. We characterized the probe's sensing properties and validated the pH-dependent shift change mechanism through theoretical calculations.

CoNO2ASF_5_ uses 1,4,7-triazacyclononane (TACN) as a macrocyclic base with two carboxylic acid arms (NO2A) and one anilide arm containing the aryl-SF_5_ moiety. This scaffold utilizes a hexa-coordinate structure for kinetic stability of the Co(ii).^26,27^ The anilide donor is key to biologically relevant pH sensitivity (compared to alkyl amide donors employed by our group) as the electron withdrawing nature of the aryl group lowers the p*K*_a_ of the anilide NH to be within the physiological pH range. We propose that anilide deprotonation results in a CoNO2ASF_5_ coordination shift (Fig. 1a), converting from the protonated, neutral, oxygen-bound species (O-bound, 1) to the deprotonated, anionic, nitrogen-bound species (N-bound, 2).

**Fig. 1:**
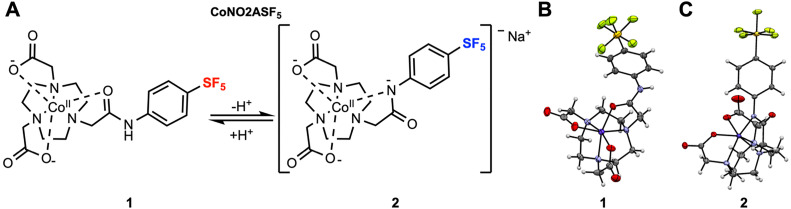
(A) Structure of CoNO2ASF_5_ before, 1, and after, 2, coordination change. Crystal structures of CoNO2ASF_5_ grown in acidic (B) and basic (C) environments with counterions omitted for clarity (carbon, gray; oxygen, red; nitrogen, light blue; sulfur, yellow; hydrogen, white; fluorine, green; and cobalt, dark purple).

Synthesis of CoNO2ASF_5_ was carried out from previously reported ^*t*^BuNO2A^13^ with chloroacetyl chloride and 4-(pentafluorothio)-aniline in two steps to afford ^*t*^BuNO2ASF_5_. Acid deprotection generated the final ligand NO2ASF_5_. Ligand NO2ASF_5_ was reacted with CoCl_2_·6H_2_O in anaerobic conditions to yield CoNO2ASF_5_. Intermediates were purified by reverse-phase chromatography and confirmed by HRMS and NMR.

Crystals of CoNO2ASF_5_ were grown as pink prisms in acidic (acetonitrile/water/HCl) and basic (methanol/water/NaOH) environments. Single crystal XRD structures demonstrate that the anilide oxygen coordinates to the cobalt in acidic conditions and the deprotonated anilide nitrogen coordinates with the cobalt in basic conditions, confirming the proposed coordination switch (Fig. 1). The coordination spheres consist of a plane of three TACN nitrogens and a plane of two carboxylate anions with the anilide. The twist angle of these planes shifts from a pseudo-octahedral angle of 41° for O-bound 1 to a less offset angle of 25° for N-bound 2. The added anionic donor in 2 is reflected by a lengthening of the Co–N_TACN_ (from 2.109 Å to 2.144 Å) and Co–O_carboxylate_ (from 2.039 Å to 2.102 Å) bond lengths after deprotonation. This is accompanied by a decrease in the deprotonated Co–N_anilide_ (2.07 Å) bond lengths compared to the protonated Co–O_anilide_ (2.123 Å) bond length. The cobalt-fluorine distance is also shortened in N-bound 2 (7.674 Å) compared to O-bound 1 (8.992 Å).

Magnetic susceptibility studies of CoNO2ASF_5_ in acidic and basic conditions confirm that both complexes are high spin *S* = 3/2 species with large orbital angular momentum contributions characteristic of high spin Co(ii), Table 1. This is reflected in the ^1^H NMR spectra of 1 and 2 which reveal the presence of highly shifted resonances ranging from −60 to +220 ppm (Fig. S2 and S3).

**Table 1:** ^19^F NMR properties of 1 mM CoNO2ASF_5_ in aq. buffer (MES,HEPES, CHES)

	^19^F_doublet_*δ* (ppm)	^19^F_doublet_ FWHM (ppm)	^19^F_doublet_*T*_1_; *T*_2_ (ms)	*T*_1_/*T*_2_	^19^F_quintet_*δ* (ppm)	*μ*_eff_
1	+66.3	13.8 ± 1.2	73.3; 2.3	31.9	+88.0	4.61 ± 0.04
2	+98.0	26.6 ± 0.4	89.0; 2.0	44.5	+93.8	4.72 ± 0.03
NO2ASF_5_	+62.9	7.2 ± 0.2	465; 328	1.42	+85.3	—

^19^F NMR characterization of 1 mM CoNO2ASF_5_ at pH 7.4 showed two doublets corresponding to the four-equivalent equatorial fluorines of the –SF_5_ moiety and two quintets corresponding to the axial fluorine. Each doublet and quintet pair could be detected in isolation by placing CoNO2ASF_5_ in acidic or basic buffers. At acidic pH (pH 5.5), the doublet and quintet peaks of complex 1 are found at +66.3 ppm and +88.0 ppm respectively. At basic pH (pH 9), the doublet and quintet peaks of deprotonated complex 2 are found at +98.0 ppm and +93.8 ppm respectively. There is an unexpectedly large >30 ppm chemical shift in the doublet peak when transitioning between the O-bound and N-bound complexes (Fig. 2A). Given the low intensity of the quintet, we will highlight the doublet peaks for all NMR samples. The doublets’ ^19^F relaxation times are significantly shortened due to the paramagnetic effect of the Co(ii), Table 1. Despite the high *T*_1_/*T*_2_ ratio and short *T*_2_, the doublet is well resolved with minimal broadening of the full-width at half maximum (FWHM) value.

**Fig. 2:**
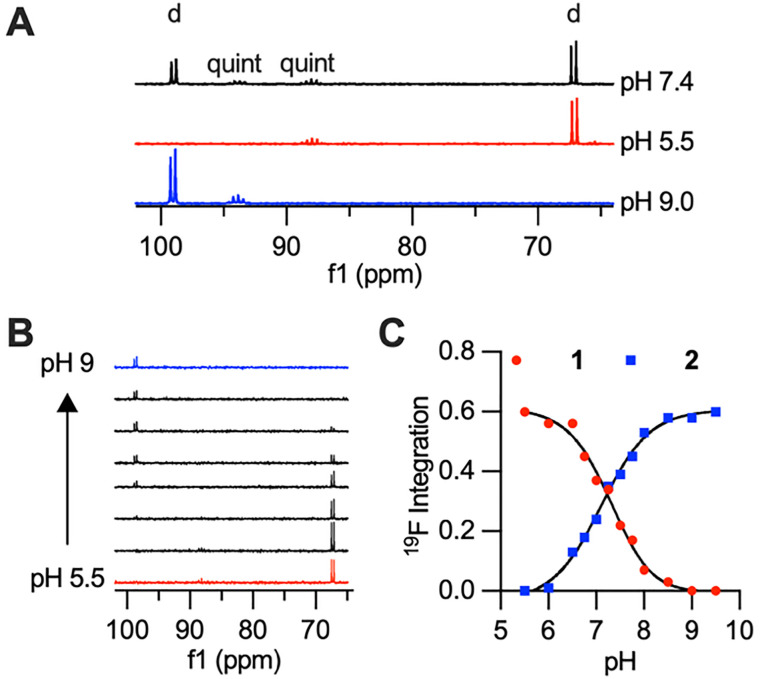
(A) ^19^F NMR spectrum of 5 mM CoNO2ASF_5_ in pH 7.4 HEPES, pH 5.5 MES, pH 9 CHES with doublets, d, and quintets, quint, labelled. (B) ^19^F NMR and (C) titration curve of CoNO2ASF_5_ in pH 5.5 – 9.5 buffers.

A pH titration of CoNO2ASF_5_ was performed to characterize the p*K*_a_ of the observed coordination change and to demonstrate that the conversion between the two ^19^F NMR doublets as pH is increased. The titration was performed using 1 mM CoNO2ASF_5_ dissolved in sulfonic acid buffers at different pH values (pH 5.0–6.5 MES, pH 7.0–8.0 HEPES, pH 8.5–9.5 CHES) and monitored by ^19^F NMR spectroscopy. Fig. 2B shows that as the pH increases, the peak at +66.3 ppm decreases and the peak at +98.0 increases. This data was fit to a p*K*_a_ of 7.2 for the anilide proton, which is firmly within the biologically relevant range. Additional cyclability tests were performed and demonstrated reversible coordination and dynamic pH monitoring by ^19^F NMR (Fig. S4).

To better understand the large change in shift between O- bound 1 and N-bound 2, NMR chemical shift calculations were performed using a hybrid protocol combining density functional theory (DFT) and *ab initio* multireference approaches.^28,29^ DFT based geometric optimization showed coordination modes consistent with their crystal structures. After deprotonation, the anilide N atom replaces the carbonyl O atom as the coordinating atom. Complete active space self-consistent field (CASSCF) calculations with N-electron valence state perturbation theory (NEVPT2) treatment of dynamic correlation validate the triplet ground states for both complexes but reveal distinct zero field splitting (ZFS) characteristics. Complex 1 exhibits a positive axial parameter *D* of +51.1 cm^−1^ (*D* = 3*D*_*zz*_/2) with *E*/*D* = 0.079 (*E* = (*D*_*xx*_ − *D*_*yy*_)/2) and *g* = [2.026, 2.481, 2.536], whereas complex 2 shows a negative *D* of −55.0 cm^−1^ with *E*/*D* = 0.142 and *g* = [2.085, 2.213, 2.766]. The calculated *g* factors well reproduce the measured effective magnetic moments (1: exp. 4.61 ± 0.04*μ*_B_, calcd 4.57*μ*_B_; 2: exp. 4.72 ± 0.03*μ*_B_, calcd 4.60*μ*_B_).

Calculated NMR chemical shifts using variant DFT functionals are summarized in Tables S5 and S6 with atom labels shown in Fig. S6. For ^19^F nuclei, the calculated values reproduce the experimental trends, Table 2. Increasing fraction of Hartree–Fock exchange in the hybrid PBE functional, from 20% to 30% (PBE-20, 20%; PBE0, 25% and PBE-30, 30%), the calculated ^19^F chemical shifts for the equatorial ^19^F in complex 2 shows slight upfield shifts, from 110.2 ppm to 108.5 ppm, while the other F sites exhibit slight downfield shifts of similar magnitude. These opposite change in chemical shifts come from the distinct magnitude of paramagnetic contributions (*σ*^p^), thus leading to their different sensitivities to the functionals used in the calculations. With PBE0 functional, the equatorial F in complex 2 (denoted 2_eq_-F) display large *σ*^p^ of *ca.* −35 ppm, while the other F nuclei only show |*σ*^p^| < 8 ppm (Tables S7 and S8). All F sites in complex 1 have positive *σ*^p^, while those in complex 2 are negative. Due to the similar Mulliken charges on F atoms of the same type, this contrast in *σ*^p^ cannot arise from anionic-fragment charge bias.

**Table 2:** Calculated NMR chemical shift for ^19^F nuclei in 1 and 2. The values are averaged values (in ppm) for all chemically equivalent nuclei

	PBE-20	PBE0	PBE-30	Exp.
1_equatorial_	67.0	67.1	69.0	66.3
2_equatorial_	110.2	109.1	108.5	98.0
1_axial_	95.6	96.2	96.9	88.0
2_axial_	105.4	107.0	108.4	93.8

Instead, Mulliken reduced orbital spin-density analysis reveals that in O-bound 1 all orbitals of all F atoms carry nearly zero spin density, whereas in N-bound 2 the axial F atom remains almost spin-neutral, while the p_*x*,*y*_ orbitals of the equatorial F atoms exhibit alpha-spin density, albeit small in magnitude (Table S9). The alpha-spin density stems from weak spin delocalization through the π conjugated pathway extending from the deprotonated amide N, which directly coordinates to Co, through the aromatic ring and S atom, and ultimately to the p_*x*,*y*_ orbitals of 2_eq_-F atoms, Fig. 3A. The alpha-spin density on p_*x*,*y*_ orbitals polarizes beta-spin density on the F 1s orbital, producing a positive hyperfine coupling through FC (*A*_iso_ of *ca.* 0.16 MHz) and a corresponding negative *σ*^p^ of *ca.* 30 ppm. This mechanism is consistent with the drastic 31.7 ppm upfield shift for the equatorial ^19^F signal after deprotonation.

**Fig. 3:**
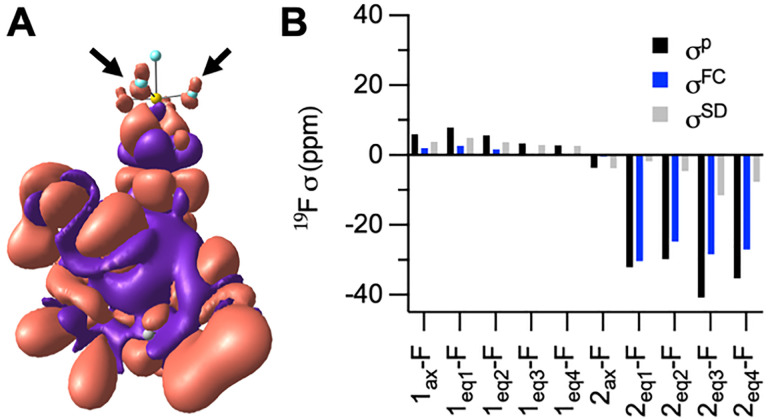
(A) DFT-calculated spin density isosurface (α spin in red and β spin in purple; isovalue = 1 × 10^−5^) overlaid on the molecular structure of complex 2. Co (dark green), S (yellow), F (light blue), N (blue), O (red), C (grey), H (light grey). (B) Calculated paramagnetic (*σ*^p^) contributions and individual contributions from Fermi contact (*σ*^FC^) and spin-dipole (*σ*^SD^) for ^19^F nuclei in complex 1 and 2, obtained using the PBE0-derived Fermi contact and spin-dipolar hyperfine terms.

The individual contributions of FC *versus* PCS were determined by extracting the FC term and the spin-dipole (SD) term of the hyperfine coupling tensor, Fig. 3B. PCS is based on the anisotropic component of the hyperfine tensor which is composed primarily of the SD term with small contributions from the gauge correction (diamagnetic) and spin-orbital coupling terms. The majority of the paramagnetic contributions to 1 come from PCS with minor to negligible FC contributions. We therefore hypothesize that the ∼4 ppm shift from the diamagnetic ligand to O-bound 1, is a result of PCS. This relatively small shift reflects the longer Co–F distance. The PCS effects in 2 are more pronounced but are insignificant compared to the large FC effects produced by the beta-spin density. Overall, these DFT results support a novel signal switching mechanism in which FC interactions are activated following deprotonation of the N-amide and coordination to Co^II^.

Given the distinct and well resolved ^19^F NMR signal, we continued with ^19^F MRI phantom studies. Selective pulse sequence imaging was utilized with optimized parameters based on the relaxation values and frequencies to image 5 mM CoNO2ASF_5_ at pH 5.5, 7.4 and 9. Pulse sequences tailored specifically to either species yielded SNR values 
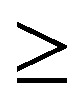
20 for both complexes in 30 minutes (Fig. 4). Each pulse sequence was selective for either the O- or N-bound complexes with the signal of the other complex below the limit of detection (SNR < 3). Both species were observed at physiological pH 7.4.

**Fig. 4:**
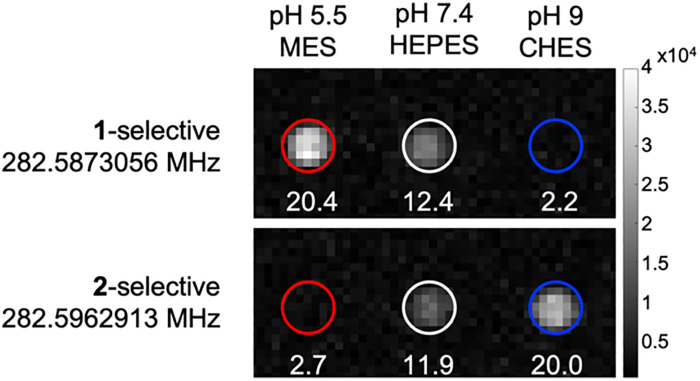
^19^F MR Phantom images of 600 µL 5 mM CoNO2ASF_5_ at pH 5.5, 7.4, and 9, scanned for 30 minutes.

In conclusion, we have synthesized and characterized a novel pH-responsive cobalt complex for ^19^F MR applications. By utilizing an anilide linker, the p*K*_a_ of the coordinating amide moiety is lowered to 7.2. This pH sensitivity is clearly observed by ^19^F MR imaging as the O- and N-bound species exhibit a 30 ppm chemical shift difference. The coordinating deprotonated anilide nitrogen allows for delocalization of spin density through a pi conjugated pathway to the fluorine atoms. This provides FC interactions to the equatorial fluorine in 2 whereas 1 has much weaker paramagnetic contributions mostly through PCS. As a new strategy for responsive PARAshift probes, this design stands out due to the exceptional shift magnitude despite a long Co–F distance. So, while many FC based paramagnetic probes exhibit strong signal quenching due to the close Co–F proximity, CoNO2ASF_5_ displays minimal signal broadening. Future work will evaluate the versatility of applying FC shifts through the extend pi system in developing sensing molecules to a variety of stimuli for MR sensing applications.

K. S. and R. K. performed experimental investigation, formal analysis, conceptualization, and writing original draft. C. H., G. E. F. B., and J. A. R. performed experimental investigation and analysis. J. X. preformed DFT and CASSCF/NEVPT2 calculations/interpretation and writing. Y. G. provided expertise, funding and editing. E. Q. provided conceptualization, formal analysis, writing, and project administration, resources and funding.

## Conflicts of interest

There are no conflicts to declare.

## Supplementary Material

CC-062-D6CC00957C-s001

CC-062-D6CC00957C-s002

## Data Availability

Data from this publication are available *via* the Texas Data Repository, https://doi.org/10.18738/T8/EI3UHt. Supplementary information (SI) is available. See DOI: https://doi.org/10.1039/d6cc00957c. CCDC 2530886 and 2530887 contain the supplementary crystallographic data for this paper.^31*a*,*b*^
