# Supplementary material for: A responsive Co(ii) 19F PARAShift probe: activation of Fermi contact interactions triggered by pH-dependent coordination changes
Source: Chem Commun (Camb). 2026 Mar 9;62(25):6741–5. doi: 10.1039/d6cc00957c (PMC12991042; doi:10.1039/d6cc00957c)
Supplement: CC-062-D6CC00957C-s001 [file CC-062-D6CC00957C-s001.pdf]

Supplementary Information (SI) for ChemComm.
This journal is © The Royal Society of Chemistry 2026

Supplementary Information

A Responsive Co(II) ^{19}F PARAShift Probe: Activation of Fermi Contact Interactions Triggered by pH-dependent Coordination Changes

Kathleen M. Scott, †[a] Rahul T. Kadakia, †[a] Christopher D. Hastings,[a] Georgia Barone,[a] Jackson Reyna,[a] Jin Xiong[b*], Yisong Guo[b] and Emily L. Que [a]

[a]Department of Chemistry, University of Texas at Austin, Austin, Texas 78712-1224, US

[b]Department of Chemistry, Carnegie Mellon University, Pittsburgh, PA 15213, US

*Corresponding authors: jinxiong@andrew.cmu.edu and emilyque@cm.utexas.edu

Experimental Details S2

Synthetic Methods S6

Scheme S1. Synthetic route for **CoNO2ASF$_5$** S6

Table S1. Selected crystallographic data for **1** S9

Table S2. Selected crystallographic data for **2** S10

Table S3. Selected distances and angles in **1** and **2** S10

Figure S1. UV-Vis spectra of 1 mM **CoNO2ASF$_5$** at acidic and basic pH S11

Table S4. ^{19}F NMR integration values from pH titration S11

Figure S2. ^{1}H NMR spectra of **1** at 25 °C and 50 °C S12

Figure S3. ^{1}H NMR spectra of **2** at 25 °C and 50 °C S13

Figure S4. Reversible pH cycling of 0.5 mM **CoNO2ASF$_5$** S14

Paramagnetic NMR Calculations Description S15

Figure S5. Optimized geometry of complexes **1** and **2** with calculated **D** and **g** frames S17

Figure S6. Geometry of complexes **1** and **2** with numeric label for individual atoms S18

Table S5. Calculated isotropic NMR shielding values of complex **1** S19

Table S6. Calculated isotropic NMR shielding values of complex **2** S20

Table S7. Calculated σ^{orb} and σ^{p} contributions to the NMR shielding tensor of **1** S21

Table S8. Calculated σ^{orb} and σ^{p} contributions to the NMR shielding tensor of **2** S22

Table S9. DFT-calculated Mulliken reduced orbital spin populations on s and p orbitals S23

Table S10. Calculated σ^{p} from σ^{FC} and σ^{SD} to the NMR shielding tensor (δ) for **1** S24

Table S11. Calculated σ^{p} from σ^{FC} and σ^{SD} to the NMR shielding tensor (δ) for **2** S24

Figure S7-9. High resolution MS for **^{t}BuNO2ASF$_5$, NO2ASF$_5$, CoNO2ASF$_5$** S25-26

Figure S10-16. NMR spectra for **^{t}BuNO2ASF$_5$, NO2ASF$_5$, CoNO2ASF$_5$** S27-33

Experimental Details

General

All solvents and chemicals were purchased from Sigma-Aldrich and Fisher Scientific and used as received. Reverse phase C18 chromatography was performed on a Biotage Isolera One. All water used in synthesis and purification was Milli-Q grade water. ^{1}H, ^{13}C, and ^{19}F NMR spectroscopic measurements were conducted in deuterated solvents purchased from Cambridge Isotope Laboratories (Cambridge, MA), using an AGILENT MR 400 NMR spectrometer at 400, 100, 376 MHz, respectively. The chemical shifts for ^{1}H and ^{13}C NMR were calibrated to the solvent peak and ^{19}F NMR was calibrated to $CFCl_3$ (δ = 0 ppm). LC-MS and high-resolution Electrospray Ionization (ESI) mass spectral analyses were performed by the Mass Spectrometry Facility of the Department of Chemistry at UT Austin. UV-vis spectra were measured on an Aligent Cary 6 UV-vis spectromenter. X-Ray crystallography was performed on two instruments: (1) an Agilent Technologies SuperNova Dual Source diffractometer using a μ-focus Cu Kα radiation source (λ = 1.5418 Å) with collimating mirror monochromators and (2) a Nonius Kappa CCD diffractometer using a Bruker AXS Apex II detector and a graphite monochromator with Mo Kα radiation (λ = 0.71073 Å).

X-ray Crystallography

Single crystal X-ray diffraction for **CoNO2ASF$_5$-1** grown in acidic environments was performed on a Nonius Kappa CCD diffractometer using a Bruker AXS Apex II detector and a graphite monochromator with Mo Kα radiation (λ = 0.71073 Å). The data was collected at 100 K using an Oxford Cryosystems 700 low-temperature device. Data reduction was performed using SAINT V8.27B. The structure was solved by direct methods using SHELXT and refined by full-matrix least-squares on F^2 with anisotropic displacement parameters for the non-H atoms using SHELXL-2016/6. Structure analysis was aided by use of the programs PLATON, OLEX2, and WinGX. The hydrogen atoms bound to carbon atoms were calculated in idealized positions.

Crystals grew as long pinkish prisms by slow evaporation from water and acetonitrile. The data crystal was cut from a larger crystal and had approximate dimensions 0.33 x 0.21 x 0.13 mm. A total of 1132 frames of data was collected using ω-scans with a scan range of 0.6° and a counting time of 63 seconds per frame. The triazacyclononane portion of the Co complex was disordered. The disorder was modeled using features available in OLEX2. The geometry of the two

components of the disorder was restrained to be equivalent. In addition, there was a region of disordered solvent located around a crystallographic inversion center. The solvent could not be adequately modeled and was removed using SQUEEZE.

The function, $\Sigma w(|F_o|^2 - |F_c|^2)^2$, was minimized, where $w = 1/[(\sigma(F_o))^2 + (0.0671*P)^2 + (0.5249*P)]$ and $P = (|F_o|^2 + 2|F_c|^2)/3$. $R_w(F^2)$ refined to 0.136, with R(F) equal to 0.0542 and a goodness of fit, S = 1.01. Definitions used for calculating R(F), $R_w(F^2)$ and the goodness of fit, S, are given below. The data was checked for secondary extinction effects, but no correction was necessary. Neutral atom scattering factors and values used to calculate the linear absorption coefficient are from the International Tables for X-ray Crystallography. All figures were generated using SHELXTL/PC. Tables of positional and thermal parameters, bond lengths and angles, torsion angles and figures are found elsewhere.

Single crystal X-ray diffraction for **CoNO2ASF$_5$-2** grown in basic environments was performed on an Agilent Technologies SuperNova Dual Source diffractometer using a μ-focus Cu Kα radiation source (λ = 1.5418 Å) with collimating mirror monochromators. The data was collected at 100 K using an Oxford Cryosystems 700 low-temperature device. Data collection, unit cell refinement, and data reduction were performed using Rigaku Oxford Diffraction's CrysAlisPro V 1.171.40.53. The structure was solved by direct methods using SHELXT and refined by full-matrix least-squares on F^2 with anisotropic displacement parameters for the non-H atoms using SHELXL-2016/6. Structure analysis was aided by use of the programs PLATON, OLEX2, and WinGX. The hydrogen atoms on the carbon atoms were calculated in ideal positions with isotropic displacement parameters set to 1.2xUeq of the attached atom (1.5xUeq for methyl hydrogen atoms).

Crystals grew as long pinkish prisms by slow evaporation from water and methanol. The data crystal was cut from a larger crystal and had approximate dimensions 0.29 x 0.18 x 0.13 mm. A total of 3092 frames of data was collected using ω-scans with a scan range of 1° and a counting time of 5 seconds per frame for frames collected with a detector offset of ± 41.6° and 18 seconds per frame with frames collected with a detector offset of 107.1°.

The function, $\Sigma w(|F_o|^2 - |F_c|^2)^2$, was minimized, where $w = 1/[(\sigma(F_o))^2 + (0.0897*P)^2 + (0.6473*P)]$ and $P = (|F_o|^2 + 2|F_c|^2)/3$. $R_w(F^2)$ refined to 0.131, with R(F) equal to 0.04826 and a goodness of fit, S = 1.04. Definitions used for calculating R(F), $R_w(F^2)$ and the goodness of fit, S, are given below. The data was checked for secondary extinction effects, but no correction was

necessary. Neutral atom scattering factors and values used to calculate the linear absorption coefficient are from the International Tables for X-ray Crystallography. All figures were generated using SHELXTL/PC. Tables of positional and thermal parameters, bond lengths and angles, torsion angles and figures are found elsewhere.

$R_w(F^2) = [\Sigma w(|F_o|^2 - |F_c|^2)^2/\Sigma w(|F_o|)^4]^{1/2}$ where w is the weight given for each reflection, $R(F) = \Sigma(|F_o| - |F_c|)/\Sigma(|F_o|)$ for reflections with $F_o > 4\ (\sigma(F_o))$, and $S = [\Sigma w(|F_o|^2 - |F_c|^2)^2/(n - p)]^{1/2}$ where n is the number of reflections and p is the number of refined parameters.

Determination of Magnetic Moment

Evan's method was used to determine the magnetic moment of **1** and **2**. ^{1}H NMR spectra were recorded of a coaxial inset in acidic conditions and basic conditions. Each sample in the coaxial insert contained 4 mM **CoNO2ASF$_5$**, 3 mM NaTFA, and 5% v/v tert-butanol in either 50 mM MES pH 5.5 prepared in D_2O or 50 mM HEPES pH 8.6 prepared in D_2O. The outer solution contained 5% v/v tert-butanol in either 50 mM MES pH 5.5 prepared in D_2O or 50 mM HEPES pH 8.6 prepared in D_2O. The NaTFA was included as an internal reference and ^{19}F NMR of each sample was preformed to determine the exact **CoNO2ASF$_5$** concentration. The gram susceptibility (χ_g) is calculated using the equation below.

$$\chi_g = (-3\Delta_f)/(4\pi fm) + \chi_0 + [\chi_0\ (d_0\text{-}d_s)]/m$$

Δ_f is the frequency difference (Hz) between the tert-butanol peak in the outer tube minus inner tube; f is the spectrometer frequency (Hz); m is the mass of Co complex per mL; and χ_0 is the mass susceptibility of D_2O ($\chi_0 = -0.6466 \times 10^{-6}$ cm^3/g). The last term in the first equation is neglected.

$$\mu_{eff} = 2.84\ (\chi_m\ T)^{1/2}$$

The molar susceptibility (χ_m) can be calculated by multiplying the gram susceptibility by complexes' molar weight and the effective magnetic moment can be calculated using equation above; T is temperature (K). The experiment was repeated two times and averaged.

^{19}F Relaxation Time Determination

T_1 and T_2 values were measured with an Agilent VNMRS 600 spectrometer using inversion-recovery sequence and Carr-Purcell-Meiboom-Gill (CPMG) sequence, respectively.

The 90° pulse was calibrated for each sample individually. Two samples were prepared in de-O_2 media such that the first one had 1 mM **$CoNO2ASF_5$** with 10% D_2O in 5 mM pH 5.5 MES and the second one had 1 mM **CoNO2ASF**$_5$ with 10% D_2O in 5 mM pH 9 CHES for the acidic and basic relaxation time, respectively. ^{19}F MR signals were observed at 18 different time points after excitation and the signal integrations were fitted to first-order exponential growth curve for T_1 measurement and first-order exponential decay for T_2 measurement.

pH Titration experiment

1 mM **$CoNO2ASF_5$** was placed 5 mM buffer (pH 5.5 MES, pH 6 MES, pH 6.5 MES, pH 7.0 HEPES, pH 7.5 HEPES, pH 8 HEPES, pH 8.5 CHES, and pH 9 CHES) at room temperature. ^{19}F NMR spectra were taken of each sample. An area under the curve function was executed in Prism to determine the total integration of each doublet. These integration values were then fit to an Asymmetric Sigmoidal, 5PL, X is log(concentration) function to determine the pK_a.

pH cycling

^{19}F NMR was recorded of 0.5 mM **$CoNO2ASF_5$** in milliQ water pHed to 10 using NaOH. Five successive additions of 40 mM HCl/ NaOH were added to by increasing volumes (5-20 µL) until the resulting solution was either pH 3 or pH 10 (**$CoNO2ASF_5$** concentrations were corrected with the increases in the volume) and observed by ^{19}F NMR.

^{19}F MRI

^{19}F MR images were obtained on a Bruker BioSpin (Karlsruhe, Germany) Pharmascan 70/16 magnet with a BioSpec two-channel console and BGA-9s gradient coil. The RF coil was tuned to the corresponding to corresponding resonant frequency of ^{19}F at 7.0 T, 282.2 MHz, using a Morris frequency sweeper (Morris Instruments, Inc. Ottawa, Ontario, Canada) while the complementary element was connected to the receive chain of the instrument. Method and sequences from ParaVision 6.0.1 (Bruker, vide supra) were utilized for imaging.
A custom printed 9-sample holder made for standard NMR tubes was used to image all samples simultaneously. Phantom images were taken of 5 mM **$CoNO2ASF_5$** in 600 µL of 50 mM MES pH 5.5, HEPES pH 7.4 or CHES pH 9 using optimized turboRARE (Rapid Acquisition with Relaxation Enhancement) sequence parameters. Parameters for imaging the acidic complex **1**:

TE (Echo Time) = 19.97 ms, TR (Repetition Time) = 150 ms, NA (Number of Averages) = 750, FA (Flip Angle) = 90º, rare factor = 4, echo spacing= 9.986 ms, BW (Bandwidth) = 10 kHz, working frequency= 282.5873056 MHz, matrix size = 64 x 64, FOV (Field of View) = 50 x 50 mm, ST (Slice Thickness) = 60 mm, scan time = 30 min]. Parameters for imaging the basic complex **2**: TE= 19.97 ms, TR= 150 ms, NA = 750, FA = 90º, rare factor = 4, echo spacing= 9.986 ms, BW = 10 kHz, working frequency= 282.5962913 MHz, matrix size = 64 x 64, FOV = 50 x 50 mm, ST = 60 mm, scan time = 30 min].

Synthetic methods

Scheme S1. Synthetic route for **CoNO2ASF$_5$**. **^{t}BuNO2A** was synthesized from previously reported literature.[1]

^{t}BuNO2ASF$_5$

K_2CO_3 (125 mg, 2.05 eq) and 4-(pentafluorosulfanyl)aniline (101.6 mg, 1.05 eq) were added to 10 mL acetone. Then, a solution containing chloroacetyl chloride (42 µL, 1.2 eq) in 5 mL acetone was added dropwise to the first mixture. The reaction was stirred at room temperature for four hours before quenching with 15 mL Milli-Q H_2O. The desired intermediate product was extracted using ethyl acetate (3X; 30 mL each), washed with brine (3X; 30 mL each), dried over Na_2SO_4, and transferred to a flame dried two-neck flask before evaporating to dryness. Then, **^{t}BuNO2A**[13] (158 mg, 1 eq), KI (73 mg, 1 eq), and K_2CO_3 (122.5 mg, 2 eq) were added to the flask. The flask was connected to a condenser, sealed, and the air was replaced with N_2 before

adding 5 mL dry MeCN. The reaction was heated to 80 °C for thirty minutes before adding the intermediate product and reacting overnight. The reaction was cooled, filtered, and subjected to C18 reverse phase chromatography. The desired product was purified using a 5% MeCN/95% H2O/0.1% formic acid to 100% MeCN/0.1% formic acid gradient (12 minute LC/MS Rt: 7.1 min). The product was isolated using 62% MeCN/38% H2O/0.1% formic acid. The product was lyophilized to remove solvents to obtain 195.4 mg **^{t}BuNO2ASF$_5$** (72%). Full NMR spectra can be viewed in **S10-S12**. ^{1}H NMR (400MHz, d_6-DMSO, 25 °C): δ 10.59 (s, 1H), δ 7.88 (d, 2H), δ 7.78 (d, 2H), δ 3.92 (s, 2H), δ 3.67 (d, 4H), δ 2.86-3.18 (m, 12H), δ 1.43 (s, 18H). ^{13}C NMR (125 MHz, d_6-DMSO, 25 °C): δ 169.20 (s), δ 167.57 (s), δ 147.45 (m), δ 141.64 (s), δ 126.87 (s), δ 119.00 (s), δ 81.26 (s), δ 56.57 (s), δ 54.98 (s), δ 49.83 (s), δ 48.77 (s), δ 48.33 (s), δ 27.78 (s). ^{19}F NMR (376 MHz, d_6-DMSO, 25 °C): δ 88.5 (quint, J=150.9), δ 64.8 (d, J=150.5). HR ESI-MS (ESI$^+$, MeOH): calculated for $[C_{26}H_{41}F_5N_4O_5S + H]^+$ 617.2791, found 617.2804. Full ESI$^+$ HRMS can be viewed in **S7.**

NO2ASF$_5$

^{t}BuNO2ASF$_5$ (200 mg, 1 eq) was transferred to a scintillation vial and 2 mL of a 1:1 TFA:$CHCl_3$ solution was added and allowed to react overnight at room temperature. The next morning, the solvent was removed and the dried crude product was suspended in 1 mL Milli-Q H_2O before directly injecting into a C18 reverse phase chromatography column. The desired product was purified using a 5% MeCN/95% H_2O/0.1% formic acid to 100% MeCN /0.1% formic acid gradient (12 minute LC/MS R_t: 4.7 min). The product was isolated using at 40% MeCN/60% H_2O/0.1% formic acid. The product was lyophilized to remove solvents to obtain 128 mg **NO2ASF$_5$** (78%). Full NMR spectra can be viewed in **S13-S15**. ^{1}H NMR (400MHz, d_6-DMSO, 25 °C): δ 10.90 (s, 1H), δ 8.05 (d, 2H), δ 7.81 (d, 2H), δ 3.48 (s, 2H), δ 3.44 (s, 4H), δ 3.11 (s, 4H), δ 2.96 (t, 4H), δ 2.67 (t, 4H). ^{13}C NMR (125 MHz, d_6-DMSO, 25 °C): δ 170.01 (s), δ 169.19 (s), δ 163.10 (s), δ 147.10 (m), δ 142.53 (s), δ 126.36 (s), δ 119.21 (s), δ 61.91 (s), δ 56.57 (s), δ 49.90 (s), δ 48.88 (s), δ 48.15 (s). ^{19}F NMR (376 MHz, d_6-DMSO, 25 °C): δ 88.92 (quint, J=151.0), δ 65.0 (d, J=150.5). HR ESI-MS (ESI$^+$, MeOH): calculated for $[C_{18}H_{25}F_5N_4O_5S + H]^+$ 505.1539, found 505.1552. Full ESI$^+$ HRMS can be viewed in **S8.**

CoNO2ASF$_5$

This reaction was carried out in an anaerobic glovebox containing de-O_2 solvents. **NO2ASF$_5$** (41 mg, 1 eq) was dissolved in 1 mL de-O_2 Milli-Q H_2O. Then, $CoCl_2{\cdot}6H_2O$ (23.6 mg, 1.2 eq) and enough de-O_2 MeCN was added to fully dissolve all compounds. If necessary, the pH was adjusted to between 5-6 with 1 M NaOH. The mixture was allowed to react at 25 °C for 3 hours until all the ligand was metalated (tracked via LC/MS). Upon completion, the solution was directly injected into a C18 reverse phase chromatography column that was primed and ran with N_2 bubbled solvents (at least two hours). The desired product was purified using 100% 50 mM NH_4OAc in Milli-Q H_2O (pH ~6.5-6.7) to 5% 50 mM NH_4OAc in Milli-Q H_2O (pH ~6.5-6.7)/95% MeCN gradient (12 minute LC/MS R_t: 3.8 min). The product was isolated using 43% MeCN/57% H_2O. The product was lyophilized to remove solvents to obtain 28.6 mg **CoNO2ASF$_5$** (63%). ^{19}F NMR (376 MHz, D_2O, 25 °C): δ 87.9 (quint, J=148.0), δ 67.0 (d, J=149.8). HR ESI-MS (ESI^+, MeOH): calculated for $[C_{18}H_{23}CoF_5N_4O_5S + H]^+$ 562.0714, found 562.0719. Full ESI^+ HRMS can be viewed in **S9.**

Table S1. Selected crystallographic data for **1**.

Empirical formula	C_{18} H_{25} Co F_5 N_4 O_6 S
Formula weight	579.41
Temperature	100.1(5) K
Wavelength	0.71073 Å
Crystal system	Monoclinic
Space group	P 1 21/c 1
Unit cell dimensions	a = 6.7293(12) Å; α = 90°<br>b = 21.385(3) Å; β = 99.130(7)°<br>c = 17.178(3) Å; γ = 90°
Volume	2440.6(7) $Å^3$
Z	4
Density (calculated)	1.577 Mg/m^3
Absorption coefficient	0.868 mm^{-1}
F(000)	1188
Crystal size	0.33 x 0.21 x 0.13 mm^3
Theta range for data collection	2.252 to 29.833°
Index ranges	-9<=h<=9, -29<=k<=29, -23<=l<=23
Reflections collected	37631
Independent reflections	6949 [R(int) = 0.0764]
Completeness to theta = 66.600°	99.9%
Absorption correction	Numerical
Max. and min. transmission	0.7459 and 0.5372
Refinement method	Full-matrix least-squares on F^2
Data / restraints / parameters	6949 / 335 / 424
Goodness-of-fit on F^2	1.027
Final R indices [I>2sigma(I)]	R1 = 0.0542, wR2 = 0.1228
R indices (all data)	R1 = 0.0914, wR2 = 0.1355
Extinction coefficient	N/A
Largest diff. peak and hole	0.739 and -0.554 e.$Å^{-3}$

Table S2. Selected crystallographic data for **2**.

Empirical formula	$C_{21} H_{34} Co F_5 N_4 Na O_8 S$
Formula weight	679.50
Temperature	100.0(2) K
Wavelength	1.54184 Å
Crystal system	Triclinic
Space group	P -1
Unit cell dimensions	a = 10.1113(4) Å; α = 64.957(4)°<br>b = 11.8024(5) Å; β = 69.304(4)°<br>c = 13.7663(5) Å; γ = 75.254(3)°
Volume	1381.63(11) $Å^3$
Z	2
Density (calculated)	1.633 Mg/m^3
Absorption coefficient	6.519 mm^{-1}
F(000)	702
Crystal size	0.29 x 0.18 x 0.13 mm^3
Theta range for data collection	3.692 to 79.369°
Index ranges	-12<=h<=12, -14<=k<=14, -16<=l<=16
Reflections collected	24532
Independent reflections	5464 [R(int) = 0.0683]
Completeness to theta = 66.600°	99.7%
Absorption correction	Gaussian and multi-scan
Max. and min. transmission	1.00000 and 0.59085
Refinement method	Full-matrix least-squares on F^2
Data / restraints / parameters	5464 / 39 / 402
Goodness-of-fit on F^2	1.038
Final R indices [I>2sigma(I)]	R1 = 0.0482, wR2 = 0.1292
R indices (all data)	R1 = 0.0510, wR2 = 0.1314
Extinction coefficient	N/A
Largest diff. peak and hole	0.620 and -0.470 e.$Å^{-3}$

Table S3. Selected distances and angles in **1** and **2**

	**1**	**2**
**Co-N_{TACN}**	2.109 ± 0.03 Å	2.144 ± 0.02 Å
**Co-$O_{carboxylate}$**	2.031, 2.047 Å	2.146, 2.058 Å
**Co-O_{ftag}**	2.123 Å	2.07 Å
**Co-$F_{equitorial}$**	8.920± 0.5 Å	7.674± 0.3 Å
**Co-F_{axial}**	10.18 Å	8.996 Å
**Twist angle**	40.9 ± 0.5	25.2± 0.5

Figure S1. UV-Vis spectra of 1 mM **CoNO2ASF$_5$** in 50 mM buffer [λ_{max}-**1**= 508 nm, λ_{max}-**2**= 502 nm].

Table S4. ^{19}F NMR integration values from pH titration.

**pH**	**1** ^{19}F Integration	**2** ^{19}F Integration
**5.5**	0.6	0
**6**	0.56	0.01
**6.5**	0.56	0.13
**6.75**	0.45	0.18
**7**	0.37	0.24
**7.25**	0.34	0.35
**7.5**	0.22	0.39
**7.75**	0.17	0.45
**8**	0.07	0.53
**8.5**	0.03	0.58
**9**	0	0.58
**9.5**	0	0.6

Figure S2. ^{1}H NMR spectra of **1** at 25 °C (bottom) and 50 °C (top).

Figure S3. ^{1}H NMR spectra of **2** at 25 °C (bottom) and 50 °C (top).

Figure S4. ^{19}F NMR monitoring of reversible pH cycling of 0.5 mM **CoNO2ASF$_5$**.

Paramagnetic NMR Calculations

Theory

Detailed theoretical background can be found in Refs.[2,3] The NMR chemical shielding tensor for paramagnetic species consists of two parts (Eq. 1): the orbital contribution($\boldsymbol{\sigma}^{orb}$) and the paramagnetic contribution($\boldsymbol{\sigma}^{p}$). For high-spin Co(II) species, magnetism can be described by a conventional effective spin Hamiltonian (Eq.2). Accordingly, the expression for $\boldsymbol{\sigma}^{p}$ can be derived (Eq.3). In solution NMR measurements, the chemical shift of the nucleus *K* is given by the isotropic value of its NMR shielding tensor, referenced to that of the reference nucleus (Eq. 4).

$$\sigma = \sigma^{orb} + \sigma^{p} \text{ (Eq. 1)}$$

$$H = S \cdot D \cdot S + \beta_e S \cdot g \cdot B + S \cdot A \cdot I \text{ (Eq. 2)}$$

where ***S***: electron spin operator; ***D***: zero-field splitting (ZFS) tensor; β_e: Bohr magneton; g: electronic ***g***-tensor; ***B***: external magnetic field vector; ***A***: hyperfine coupling tensor; ***I***: nuclear spin operator.

$$\sigma^{p} = -\frac{\beta_e S(S+1)}{g_N \beta_N 3kT} g \cdot Z \cdot A \text{ (Eq.3)}$$

where ***Z***: a dimensionless 3x3 matrix with matrix element defined as

$$Z_{ij} = \frac{3}{S(S+1)} \frac{1}{\sum_{\lambda',a} e^{-E_\lambda/kT}} \sum_{\lambda} e^{-E_\lambda/kT} \Big[\sum_{a,a'} < S\lambda a|S_i|S\lambda a' > < S\lambda a'|S_i|S\lambda a > + 2kT \sum_{\lambda' \neq \lambda} \sum_{a,a'} \frac{< S\lambda a|S_i|S\lambda}{}$$ [illegible]

$$\Big], \ (i,j = x,y,z)$$

$$\delta_K = \sigma_{ref} - \sigma_K \text{ (Eq.4)}$$

where $\sigma_i = \frac{1}{3} Tr(\sigma_i)$

Methods

The NMR chemical shift calculations were performed using a hybrid protocol combining density functional theory (DFT) and *ab initio* multireference approaches. Molecular fragments of the complexes were extracted from crystal structures without solvent or counterions, yielding an isolated neutral Co-SF5 complex (**1**) and an isolated Co-SF5-deproVL anion (**2**). Reference molecules ($CFCl_3$ for ^{19}F, $SiMe_4$ for ^{1}H and ^{13}C) were included for chemical shift calibration. Geometric optimization and frequency analysis at DFT level ensured well-defined minima for all

fragments. For Co(II) species, the ***D*** tensor and ***g*** tensor were obtained using quasi degenerate perturbation theory (QDPT) based on complete-active-space self-consistent-field (CASSCF) calculations with n-electron valence second-order perturbation theory (NEVPT2) correction. The complete ***A*** tensors, which include the Fermi contact term ($\boldsymbol{A}_{\mathrm{FC}}$), spin dipolar term ($\boldsymbol{A}_{\mathrm{SD}}$), spin-orbital coupling term ($\boldsymbol{A}_{\mathrm{orb}}$) and gauge correction term ($\boldsymbol{A}_{\mathrm{dia}}$), were calculated at the DFT level. The $\boldsymbol{\sigma}^{\mathrm{orb}}$ tensors were computed using gauge-independent atomic orbitals (GIAO) approach, while the $\boldsymbol{\sigma}^{\mathrm{p}}$ tensors were calculated using orca_pnmr utility. The Fermi contact and spin-dipolar contributions shown in Fig. 3 were evaluated using $\boldsymbol{A}_{\mathrm{FC}}$ and $\boldsymbol{A}_{\mathrm{SD}}$, respectively, while the $\boldsymbol{A}_{\mathrm{orb}}$ and $\boldsymbol{A}_{\mathrm{dia}}$ terms are not included in this decomposition.

Geometric optimization and frequency analysis were performed using Gaussian 16 Rev. C.01 package.[4] The PBE0 functional was applied with Grimme's D3 dispersion correction (Becke-Johnson damping, GD3BJ) and the conductor-like polarizable continuum model (CPCM) for water solvent. Def2-TZVP basis sets were employed to describe all atoms. State-averaged CASSCF/NEVPT2/QDPT calculations were performed using ORCA 6.1.0.[5] The seven 3*d* electrons of Co(II), together with the five 3*d* orbitals, were included in the active space (CAS(7,5)), considering all ten quartet and forty doublet states. The initial guess was generated using polarized atomic density method with small basis sets (Def2-TZVPP for Co, while SV for the rest atoms). The resolution-of-the-identity (RI) approximation was employed in the NEVPT2 corrections. Def2-TZVPP basis sets were applied for Co and the first coordination sphere, while def2-TZVP basis sets were applied for the remaining atoms. The $\boldsymbol{\sigma}^{\mathrm{orb}}$ tensors and ***A*** tensors were calculated through high-accuracy DFT calculations using ORCA 6.1.0, where def2-TZVPP basis sets were applied to all atoms, and fine integration grids (DefGrid3) were employed. In addition to PBE0 functional, two modified-PBE0 functionals were applied for comparison in the single point calculations, varying the fraction of Hartree-Fock (HF) exchange from 25% in PBE0 to 20% (denoted PBE-20) and 30% (denoted PBE-30), respectively.

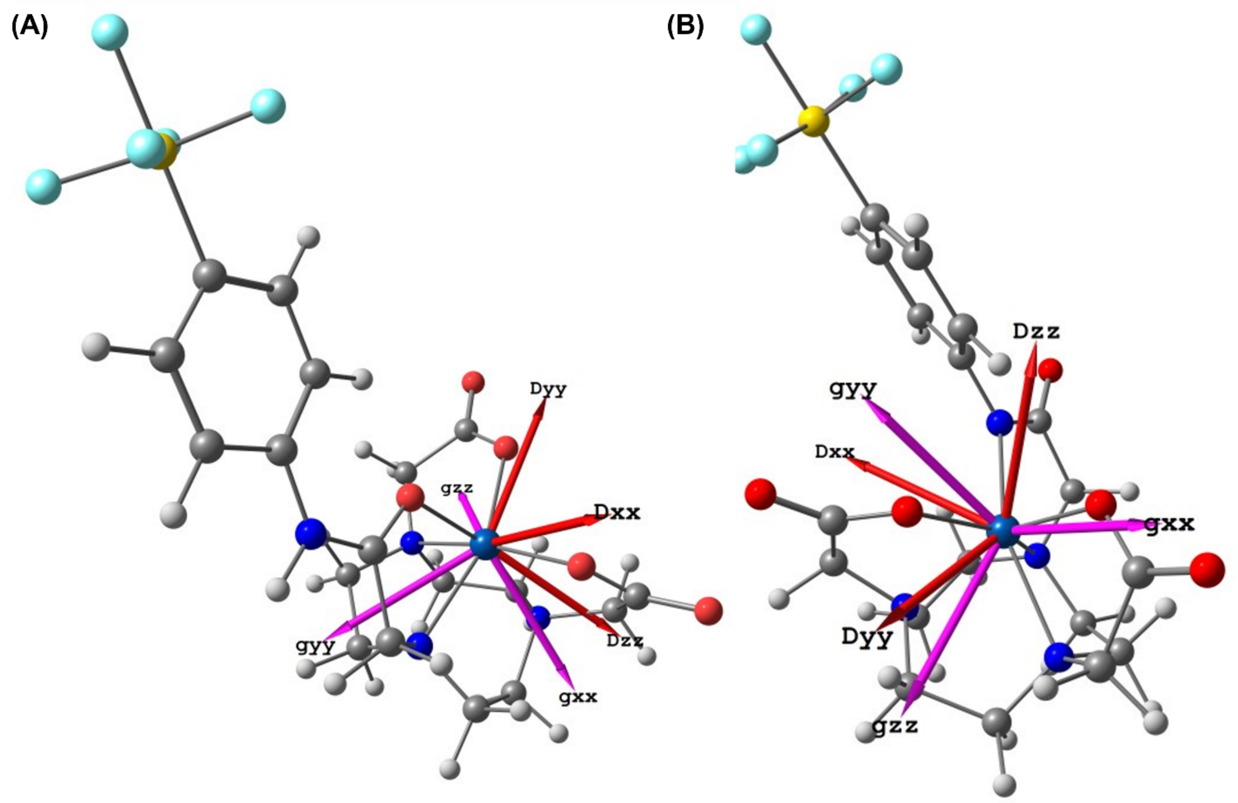

Figure S5. Optimized geometry of complexes **1** (A) and **2** (B) with calculated ***D*** and ***g*** frames. Co in dark green, S in yellow, F in light blue, N in blue, O in red, C in grey, H in light grey. Red arrows show ***D*** frames, while magenta arrows show ***g*** frames.

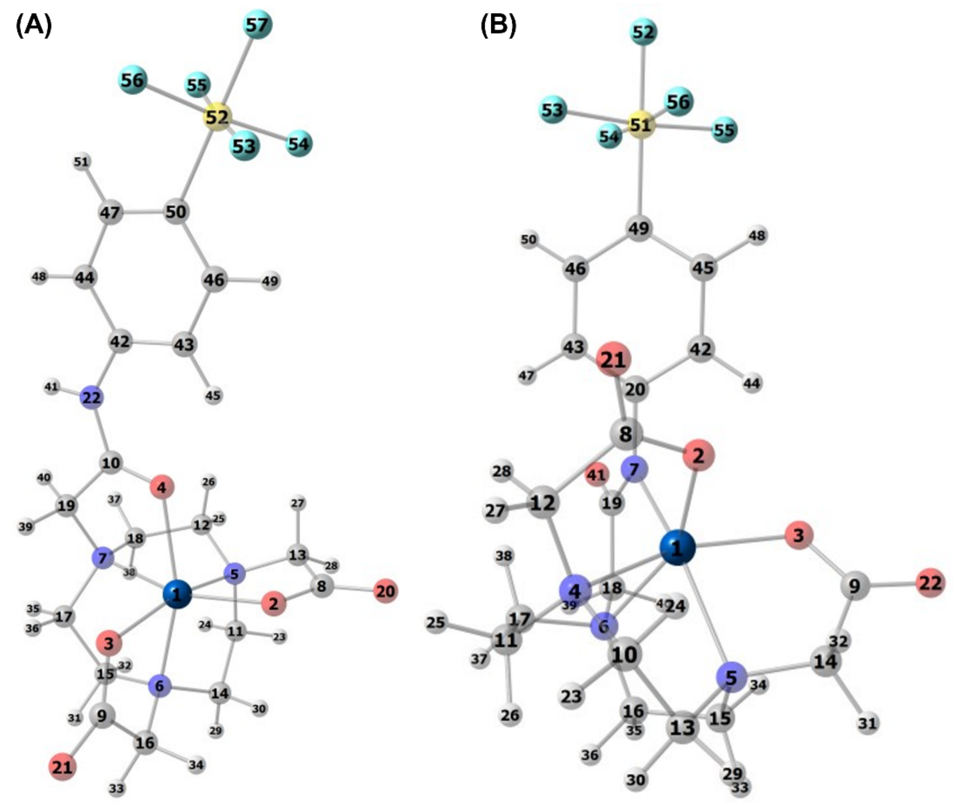

Figure S6. Optimized geometry of complexes **1** (A) and **2** (B) with numeric label for individual atoms. Co in dark green, S in yellow, F in light blue, N in blue, O in red, C in grey, H in light grey.

Table S5. Calculated isotropic NMR shielding values for reference nuclei and calculated NMR chemical shifts for the ^{1}H, ^{13}C, and ^{19}F nuclei in complex **1**. The reference values represent the average over all chemical equivalent atoms in each reference molecules.

Atom	Element	PBE-20	PBE0	PBE-30
CFCl3	F	174.025	179.823	185.429
TMS	H	31.477	31.487	31.498
TMS	C	188.772	189.516	190.242
8	C	-44.905	-18.22	-2.341
9	C	14.258	49.713	42.023
10	C	-304.05	-282.856	-281.333
11	C	-553.216	-549.337	-540.317
12	C	-444.185	-450.303	-454.019
13	C	-552.103	-536.971	-524.549
14	C	-215.934	-232.269	-234.737
15	C	-106.861	-113.63	-123.673
16	C	-548.246	-537.524	-533.108
17	C	594.954	536.31	489.813
18	C	-403.197	-385.514	-378.452
41	C	-338.515	-346.349	-353.494
42	C	160.553	157.054	155.454
43	C	119.411	116.713	113.92
45	C	114.67	113.057	112.071
46	C	139.774	138.774	138.37
49	C	134.495	134.766	135.395
22	C	150.145	148.306	147.001
23	H	122.976	111.412	102.296
24	H	61.605	59.529	58.584
25	H	195.543	175.375	158.081
26	H	-22.111	-23.229	-25.142
27	H	-32.846	-29.764	-28.521
28	H	128.828	119.096	110.764
29	H	112.045	89.037	71.575
30	H	-18.029	-19.644	-21.406
31	H	104.119	92.229	82.412
32	H	37.517	34.792	32.553
33	H	69.22	55.525	41.335
34	H	-97.159	-95.653	-96.162
35	H	224.901	202.897	185.979
36	H	57.483	56.924	55.973
37	H	127.256	116.768	108.503
38	H	72.887	70.271	69.114
39	H	44.899	48.123	50.044
40	H	144.522	127.284	114.529
44	H	74.304	67.933	62.826
47	H	-33.505	-34.444	-35.181
48	H	2.545	2.384	2.365
50	H	-3.09	-3.242	-3.35
52	H	2.901	2.745	2.678
53	F	66.818	67.082	69.034
54	F	64.657	65.067	67.076
55	F	66.95	67.085	68.974
56	F	69.483	69.348	71.101
57	F	96.201	95.553	96.913

Table S6. Calculated isotropic NMR shielding values for reference nuclei and calculated NMR chemical shifts for the ^{1}H, ^{13}C, and ^{19}F nuclei in complex **2**. The reference values represent the average over all chemical equivalent atoms in each reference molecules.

	Element	PBE20	PBE0	PBE30
CFCl3	F	174.025	179.823	185.429
TMS	H	31.477	31.487	31.498
TMS	C	188.772	189.516	190.242
8	C	-354.026	-337.752	-323.282
9	C	-548.495	-520.743	-495.94
10	C	-278.87	-264.474	-250.137
11	C	-8.516	-16.166	-20.988
12	C	-418.084	-419.824	-419.704
13	C	-454.14	-444.567	-434.287
14	C	-639.62	-633.201	-625.727
15	C	-596.302	-590.814	-583.469
16	C	-480.925	-479.743	-476.271
17	C	52.461	33.524	18.525
18	C	-603.398	-588.545	-574.509
19	C	-160.332	-144.517	-131.804
41	C	-350.059	-352.881	-356.659
42	C	521.269	508.128	498.189
44	C	550.935	532.677	517.204
45	C	73.048	73.142	72.254
48	C	108.402	103.496	98.125
22	C	278.792	280.142	282.499
23	H	183.58	163.147	146.04
24	H	-44.994	-48.229	-50.99
25	H	166.84	154.147	143.38
26	H	136.915	134.688	132.967
27	H	124.738	101.59	82.556
28	H	-53.717	-54.217	-54.711
29	H	137.244	126.955	117.991
30	H	129.345	128.109	127.175
31	H	106.485	90.446	76.68
32	H	-65.689	-66.001	-66.44
33	H	207.097	186.399	168.577
34	H	-8.023	-12.437	-16.37
35	H	129.065	117.395	107.255
36	H	107.234	106.646	106.219
37	H	270.936	251.94	235.292
38	H	3.952	1.361	-0.995
39	H	17.009	7.353	-1.038
43	H	-110.893	-111.487	-112.174
46	H	161.529	164.207	166.65
47	H	-27.421	-26.584	-26.052
49	H	46.086	46.7	47.435
51	H	26.752	26.327	26.113
52	F	105.372	106.956	108.402
53	F	107.837	106.473	105.594
54	F	105.539	104.647	104.278
55	F	116.21	114.974	114.313
56	F	111.2	110.194	109.699

Table S7. Calculated isotropic orbital (σ^{orb}) and paramagnetic (σ^{p}) contributions to the NMR shielding tensor, as well as the NMR chemical shifts (δ) for ^{1}H, ^{13}C and ^{19}F nuclei in complex **1**, obtained using the PBE0 functional. All values are in ppm.

Nucleus	Element	σ^{orb}	σ^{p}	σ	δ
8	C	6.206	201.53	207.736	-18.22
9	C	8.401	131.402	139.803	49.713
10	C	10.334	462.038	472.372	-282.856
11	C	128.973	609.88	738.853	-549.337
12	C	127.607	512.212	639.819	-450.303
13	C	118.777	607.71	726.487	-536.971
14	C	128.845	292.94	421.785	-232.269
15	C	134.129	169.017	303.146	-113.63
16	C	125.102	601.938	727.04	-537.524
17	C	128.122	-474.916	-346.794	536.31
18	C	131.682	443.348	575.03	-385.514
41	C	120.254	415.611	535.865	-346.349
42	C	42.29	-9.828	32.462	157.054
43	C	66.03	6.773	72.803	116.713
45	C	64.339	12.12	76.459	113.057
46	C	54.194	-3.452	50.742	138.774
49	C	54.512	0.238	54.75	134.766
22	C	32.064	9.146	41.21	148.306
23	H	28	-107.925	-79.925	111.412
24	H	27.025	-55.067	-28.042	59.529
25	H	28.412	-172.3	-143.888	175.375
26	H	30.274	24.442	54.716	-23.229
27	H	29.087	32.164	61.251	-29.764
28	H	28.011	-115.62	-87.609	119.096
29	H	28.552	-86.102	-57.55	89.037
30	H	28.679	22.452	51.131	-19.644
31	H	29.539	-90.281	-60.742	92.229
32	H	27.757	-31.062	-3.305	34.792
33	H	29.518	-53.556	-24.038	55.525
34	H	29.94	97.2	127.14	-95.653
35	H	28.262	-199.672	-171.41	202.897
36	H	28.863	-54.3	-25.437	56.924
37	H	28.504	-113.785	-85.281	116.768
38	H	27.184	-65.968	-38.784	70.271
39	H	26.927	-43.563	-16.636	48.123
40	H	27.37	-123.167	-95.797	127.284
44	H	23.283	-59.729	-36.446	67.933
47	H	22.818	43.113	65.931	-34.444
48	H	24.058	5.045	29.103	2.384
50	H	23.324	11.405	34.729	-3.242
52	H	23.262	5.48	28.742	2.745
53	F	106.792	5.949	112.741	67.082
54	F	106.832	7.924	114.756	65.067
55	F	107.159	5.579	112.738	67.085
56	F	107.132	3.343	110.475	69.348
57	F	81.458	2.812	84.27	95.553

Table S8. Calculated isotropic orbital (σ^{orb}) and paramagnetic (σ^{p}) contributions to the NMR shielding tensor, as well as the NMR chemical shifts (δ) for ^{1}H, ^{13}C and ^{19}F nuclei in complex **2**, obtained using the PBE0 functional. All values are in ppm.

Nucleus	Element	σ^{orb}	σ^{p}	σ	δ
8	C	10.187	517.081	527.268	-337.752
9	C	7.655	702.604	710.259	-520.743
10	C	132.615	321.375	453.99	-264.474
11	C	137.067	68.615	205.682	-16.166
12	C	126.381	482.959	609.34	-419.824
13	C	135.789	498.294	634.083	-444.567
14	C	124.657	698.06	822.717	-633.201
15	C	131.54	648.79	780.33	-590.814
16	C	136.34	532.919	669.259	-479.743
17	C	132.903	23.089	155.992	33.524
18	C	125.26	652.801	778.061	-588.545
41	C	10.37	323.663	334.033	-144.517
42	C	25.378	517.019	542.397	-352.881
43	C	53.916	-372.528	-318.612	508.128
45	C	57.539	-400.7	-343.161	532.677
46	C	56.596	59.778	116.374	73.142
49	C	57.715	28.305	86.02	103.496
22	C	36.45	-127.076	-90.626	280.142
23	H	28.97	-160.63	-131.66	163.147
24	H	30.004	49.712	79.716	-48.229
25	H	28.487	-151.147	-122.66	154.147
26	H	26.932	-130.133	-103.201	134.688
27	H	28.966	-99.069	-70.103	101.59
28	H	29.256	56.448	85.704	-54.217
29	H	28.633	-124.101	-95.468	126.955
30	H	26.897	-123.519	-96.622	128.109
31	H	28.479	-87.438	-58.959	90.446
32	H	29.109	68.379	97.488	-66.001
33	H	28.614	-183.526	-154.912	186.399
34	H	29.426	14.498	43.924	-12.437
35	H	28.705	-114.613	-85.908	117.395
36	H	26.942	-102.101	-75.159	106.646
37	H	28.743	-249.196	-220.453	251.94
38	H	29.15	0.976	30.126	1.361
39	H	28.915	-4.781	24.134	7.353
40	H	29.7	113.274	142.974	-111.487
44	H	21.389	-154.109	-132.72	164.207
47	H	23.803	34.268	58.071	-26.584
48	H	23.011	-38.224	-15.213	46.7
50	H	23.435	-18.275	5.16	26.327
52	F	76.589	-3.722	72.867	106.956
53	F	105.494	-32.144	73.35	106.473
54	F	104.947	-29.771	75.176	104.647
55	F	105.633	-40.784	64.849	114.974
56	F	104.903	-35.274	69.629	110.194

Table S9. DFT-calculated Mulliken reduced orbital spin populations on s and p orbitals for all F atoms in complexes **1** and **2**. PBE0 functionals was applied.

Complex_Atom	Mulliken Reduced Orbital Spin Populations on s and p orbitals (10^{-5} a.u.)	
**1**_F53 (eq)	s : 0.0	p_x : -0.1<br>p_y : -0.1<br>p_z : 0.1
**1**_F54 (eq)	s : 0.0	p_x : -0.1<br>p_y : -0.2<br>p_z : 0.0
**1**_F55 (eq)	s : 0.0	p_x : -0.3<br>p_y : 0.0<br>$p_{z.}$: 0.2
**1**_F56 (eq)	s : -0.0	p_x : -0.1<br>p_y : 0.2<br>$p_{z.}$: 0.1
**1**_F57 (ax)	s : -0.0	p_x : -0.0<br>p_y : -0.0<br>$p_{z.}$: -0.0
**2**_F52 (ax)	s : 0.0	p_x : -0.2<br>p_y : 0.0<br>$p_{z.}$: 0.0
**2**_F53 (eq)	s : -0.6	p_x : 3.2<br>p_y : 0.0<br>p_z : 0.1
**2**_F54 (eq)	s : -0.5	p_x : 3.4<br>p_y : 0.1<br>$p_{z.}$: -0.1
**2**_F55 (eq)	s : -0.6	p_x : 2.1<br>p_y : 1.7<br>$p_{z.}$: -0.3
**2**_F56 (eq)	s : -0.6	p_x : 1.5<br>p_y : 2.1<br>$p_{z.}$: -0.2

Table S10. Calculated paramagnetic (σ^p) contributions and individual contributions from Fermi contact (σ^{FC}) and spin-dipolar interaction (σ^{SD})and to the NMR shielding tensor, as well as the NMR chemical shifts (δ) for ^{19}F nuclei in complex **1**, obtained using the PBE0 functional. All values are in ppm.

Nucleus	Element	σ^p	σ^{FC}	σ^{SD}
53	F	5.949	1.974	3.747
54	F	7.924	2.604	4.896
55	F	5.579	1.592	3.66
56	F	3.343	0.199	2.897
57	F	2.812	-0.033	2.643

* σ^p values are calculated based on the total $\boldsymbol{A}$ tensors, where $\boldsymbol{A} = \boldsymbol{A}_{FC} + \boldsymbol{A}_{SD} + \boldsymbol{A}_{orb} + \boldsymbol{A}_{dia.}$

Table S11. Calculated paramagnetic (σ^p) contributions and individual contributions from Fermi contact (σ^{FC}) and spin-dipolar interaction (σ^{SD})and to the NMR shielding tensor, as well as the NMR chemical shifts (δ) for ^{19}F nuclei in complex **2**, obtained using the PBE0 functional. All values are in ppm.

Nucleus	Element	σ^p	σ^{FC}	σ^{SD}
53	F	-3.722	-0.451	-3.788
54	F	-32.144	-30.272	-1.775
55	F	-29.771	-24.773	-4.628
56	F	-40.784	-28.4	-11.531
57	F	-35.274	-27.012	-7.634

* σ^p values are calculated based on the total $\boldsymbol{A}$ tensors, where $\boldsymbol{A} = \boldsymbol{A}_{FC} + \boldsymbol{A}_{SD} + \boldsymbol{A}_{orb} + \boldsymbol{A}_{dia.}$

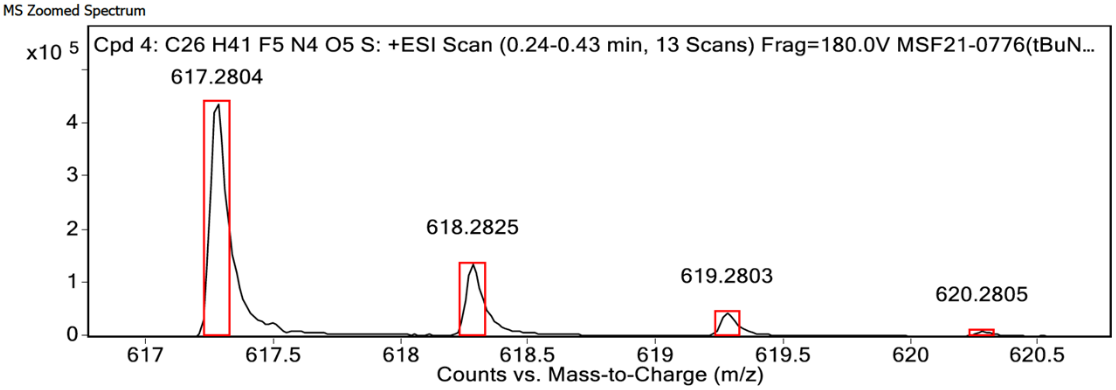

MS Spectrum Peak List

Obs. m/z	Calc. m/z	Charge	Abundance	Formula	Ion Species	Tgt Mass Error (ppm)
617.2804	617.2791	1	441320	C26H41F5N4O5S	(M+H)+	-2.14
618.2825	618.2821	1	135168	C26H41F5N4O5S	(M+H)+	-0.71
619.2803	619.2804	1	43252	C26H41F5N4O5S	(M+H)+	0.22
620.2805	620.2814	1	8442	C26H41F5N4O5S	(M+H)+	1.36
621.2794	621.2827	1	1589	C26H41F5N4O5S	(M+H)+	5.39
622.3420	622.2844	1	419	C26H41F5N4O5S	(M+H)+	-92.52
623.2817	623.2863	1	241	C26H41F5N4O5S	(M+H)+	7.38

--- End Of Report ---

Figure S7. ESI$^+$ HRMS of [^{t}**BuNO2ASF$_5$** + H]$^+$.

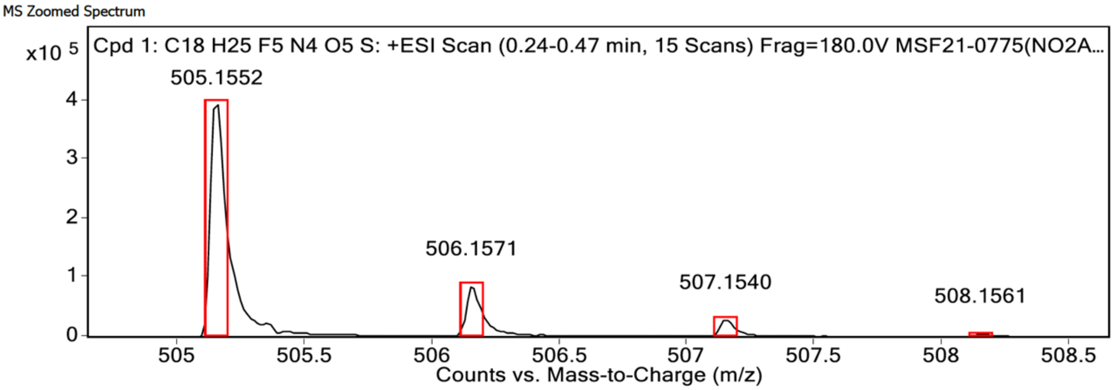

MS Spectrum Peak List

Obs. m/z	Calc. m/z	Charge	Abundance	Formula	Ion Species	Tgt Mass Error (ppm)
505.1552	505.1539	1	398389	C18H25F5N4O5S	(M+H)+	-2.6
506.1571	506.1567	1	86030	C18H25F5N4O5S	(M+H)+	-0.8
507.1540	507.1537	1	29122	C18H25F5N4O5S	(M+H)+	-0.64
508.1561	508.1552	1	5831	C18H25F5N4O5S	(M+H)+	-1.73
509.1558	509.1564	1	1528	C18H25F5N4O5S	(M+H)+	1.13
510.1682	510.1581	1	398	C18H25F5N4O5S	(M+H)+	-19.81

--- End Of Report ---

Figure S8. ESI$^+$ HRMS of [**NO2ASF$_5$** + H]$^+$.

MS Zoomed Spectrum

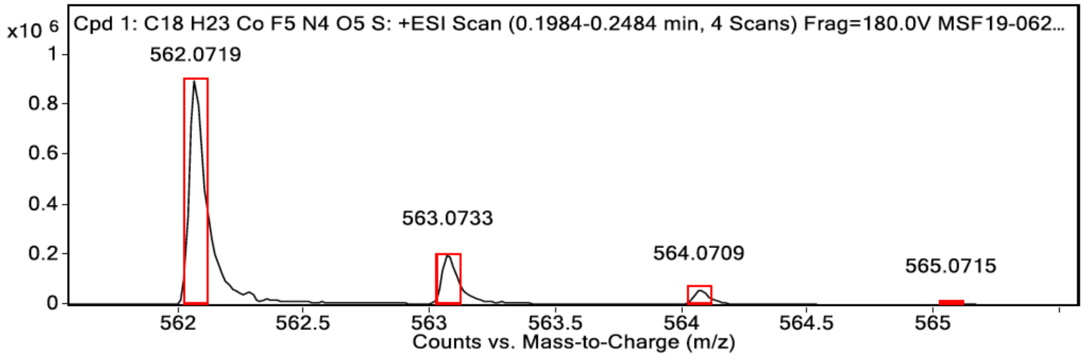

MS Spectrum Peak List

Obs. m/z	Calc. m/z	Charge	Abundance	Formula	Ion Species	Tgt Mass Error (ppm)
562.0719	562.0714	1	900288	C18H23CoF5N4O5S	(M+H)+	-0.9
563.0733	563.0742	1	202917	C18H23CoF5N4O5S	(M+H)+	1.62
564.0709	564.0713	1	62707	C18H23CoF5N4O5S	(M+H)+	0.56
565.0715	565.0727	1	11549	C18H23CoF5N4O5S	(M+H)+	2.19
566.0728	566.0739	1	1656	C18H23CoF5N4O5S	(M+H)+	1.95

--- End Of Report ---

Figure S9. ESI$^+$ HRMS of [**CoNO2ASF$_5$** + H]$^+$.

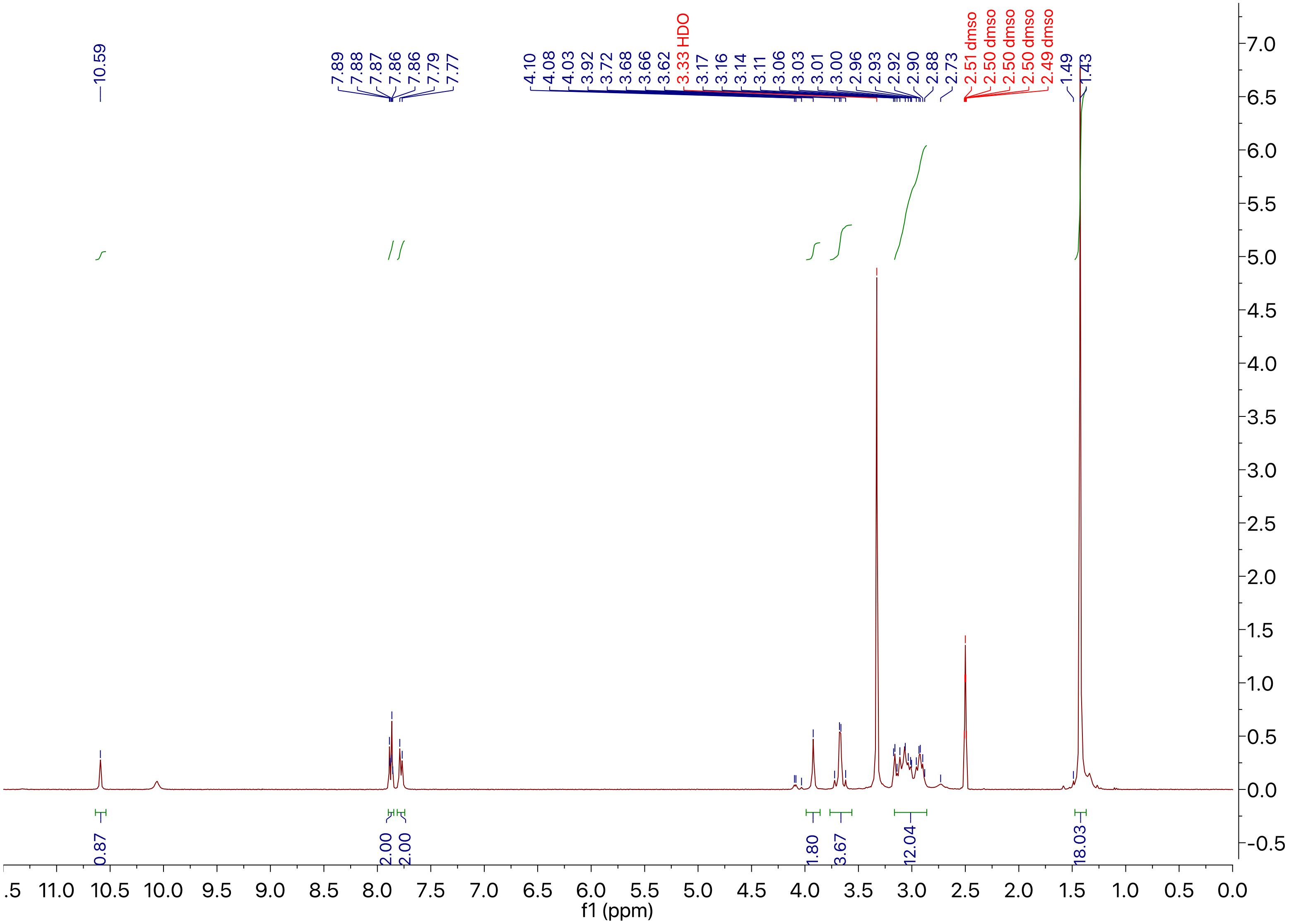

Figure S10.^{1}H NMR of ^{t}**BuNO2ASF$_5$** in d_6-DMSO at 25 °C.

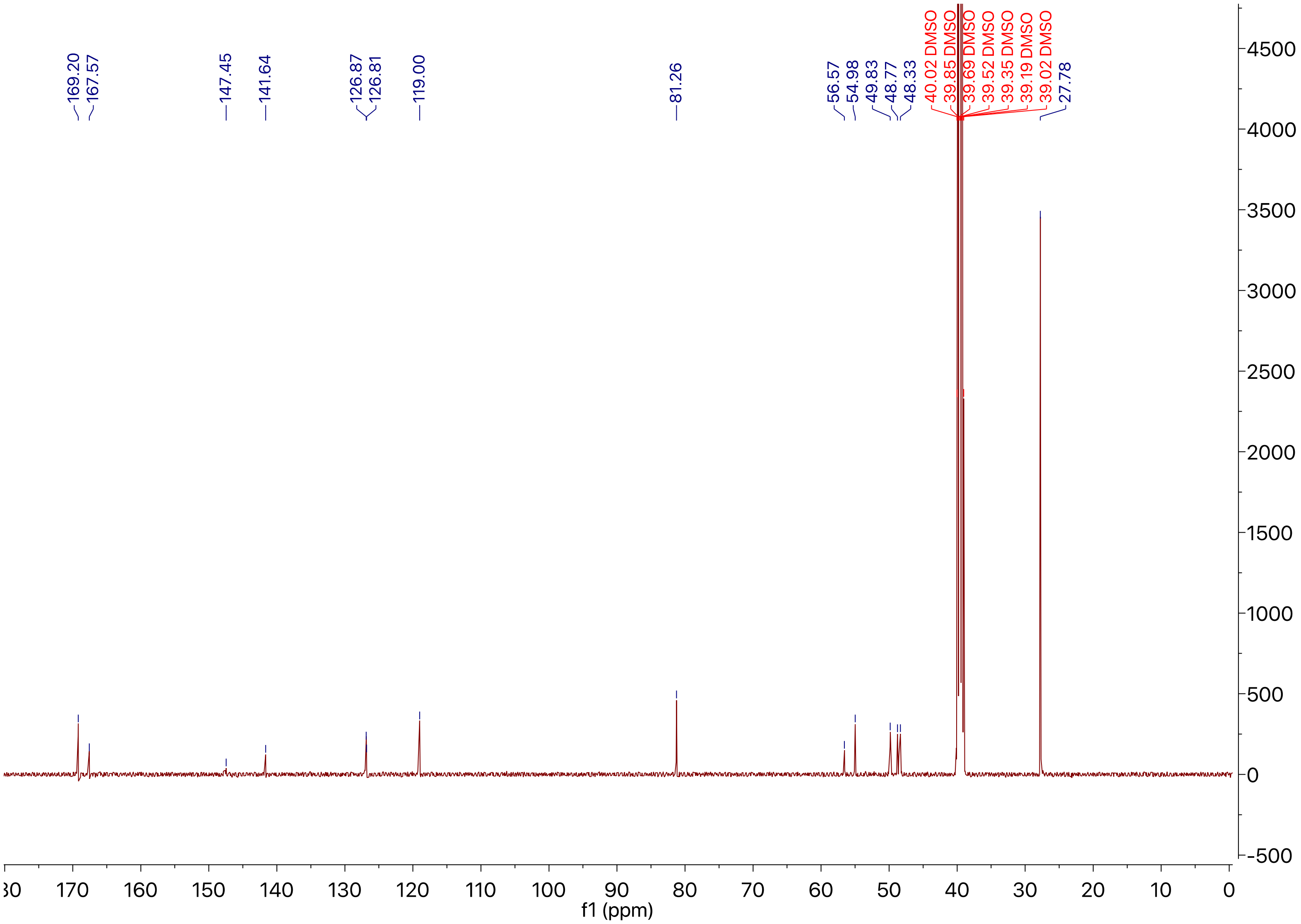

Figure S11. ^{13}C NMR of ^{t}**BuNO2ASF$_5$** in d$_6$-DMSO at 25 °C. Sample contains methanol.

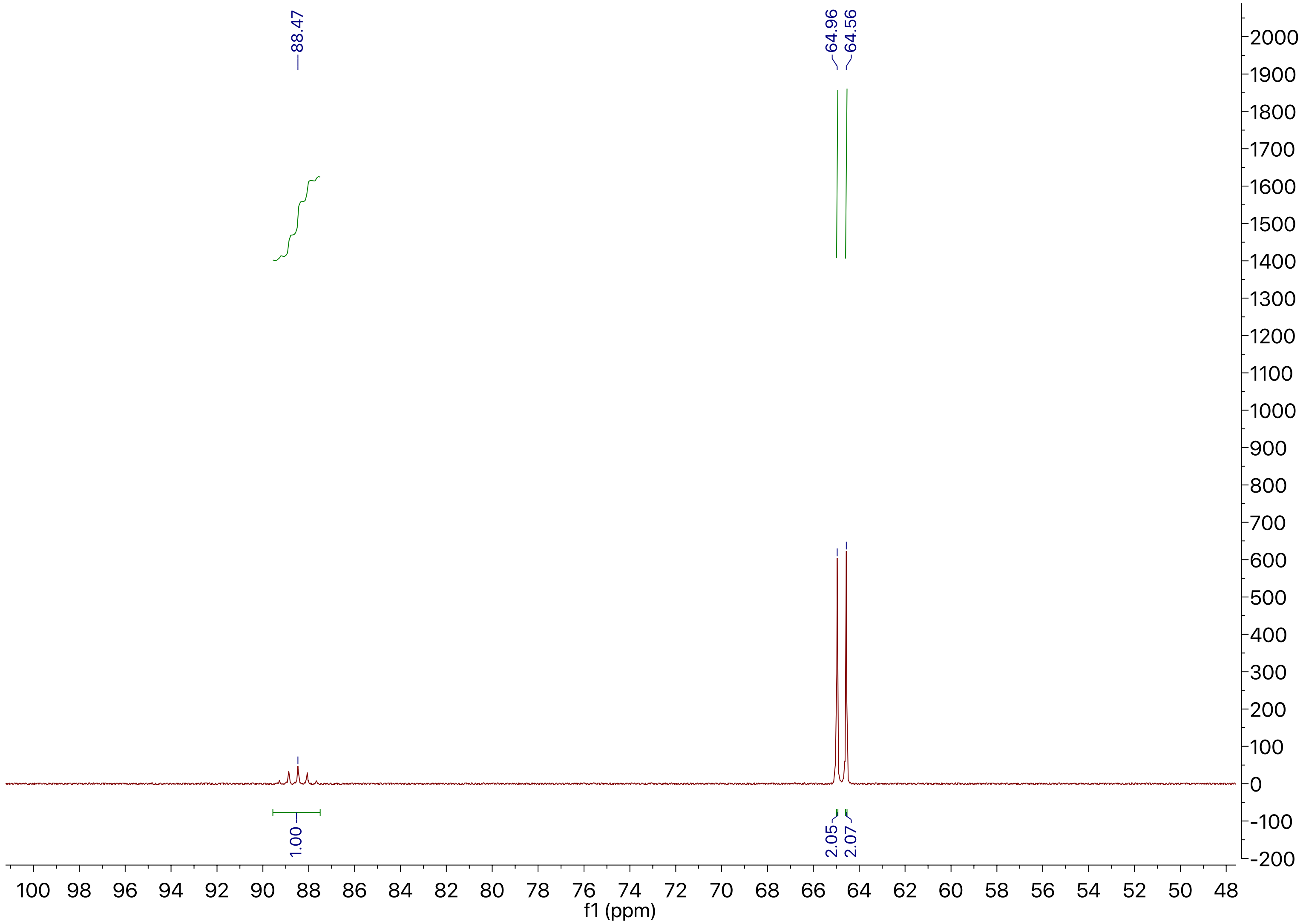

Figure S12. ^{19}F NMR of **^{t}BuNO2ASF$_5$** in d_6-DMSO at 25 °C.

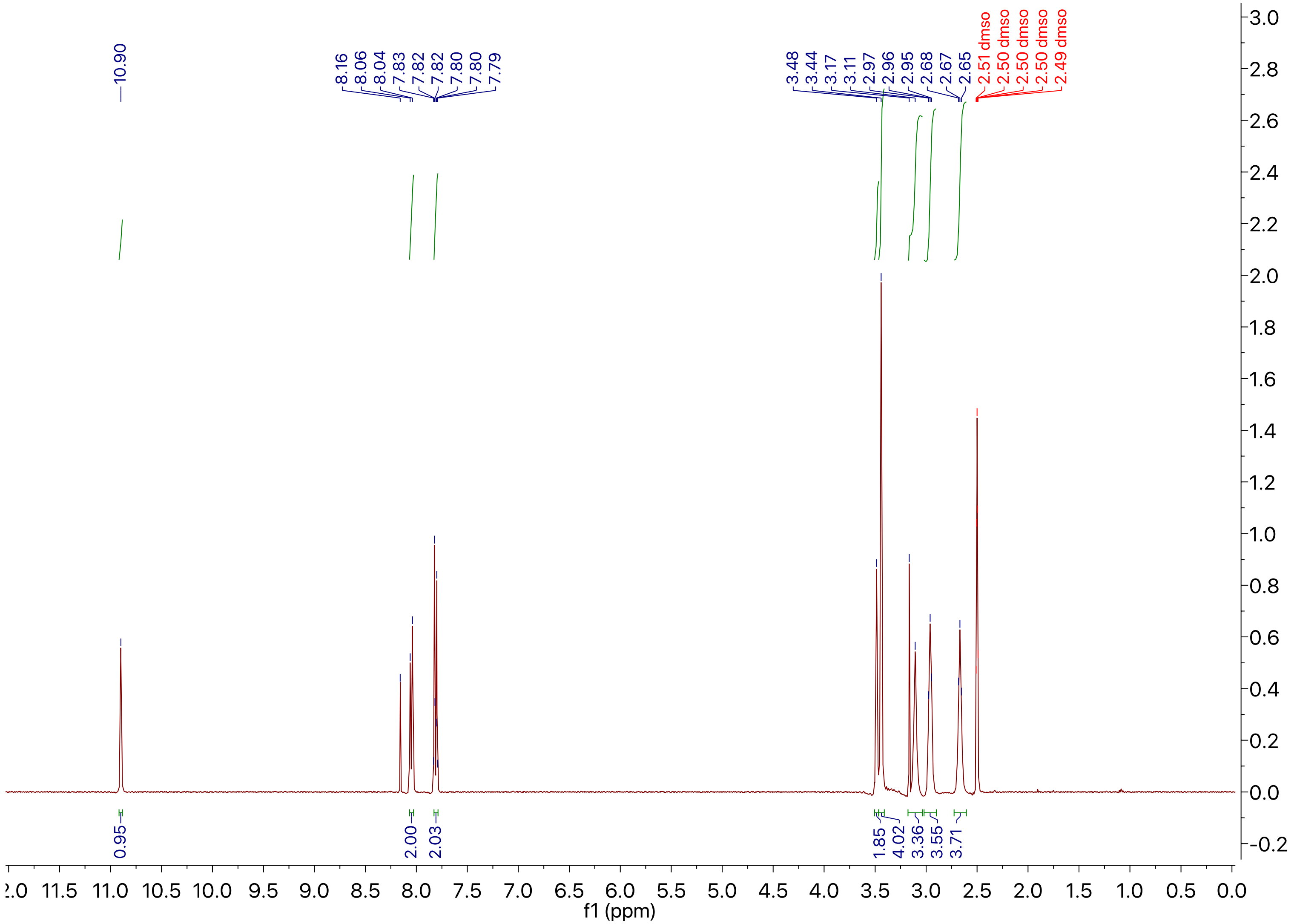

Figure S13. ^{1}H NMR of **NO2ASF$_5$** in d_6-DMSO at 25 °C.

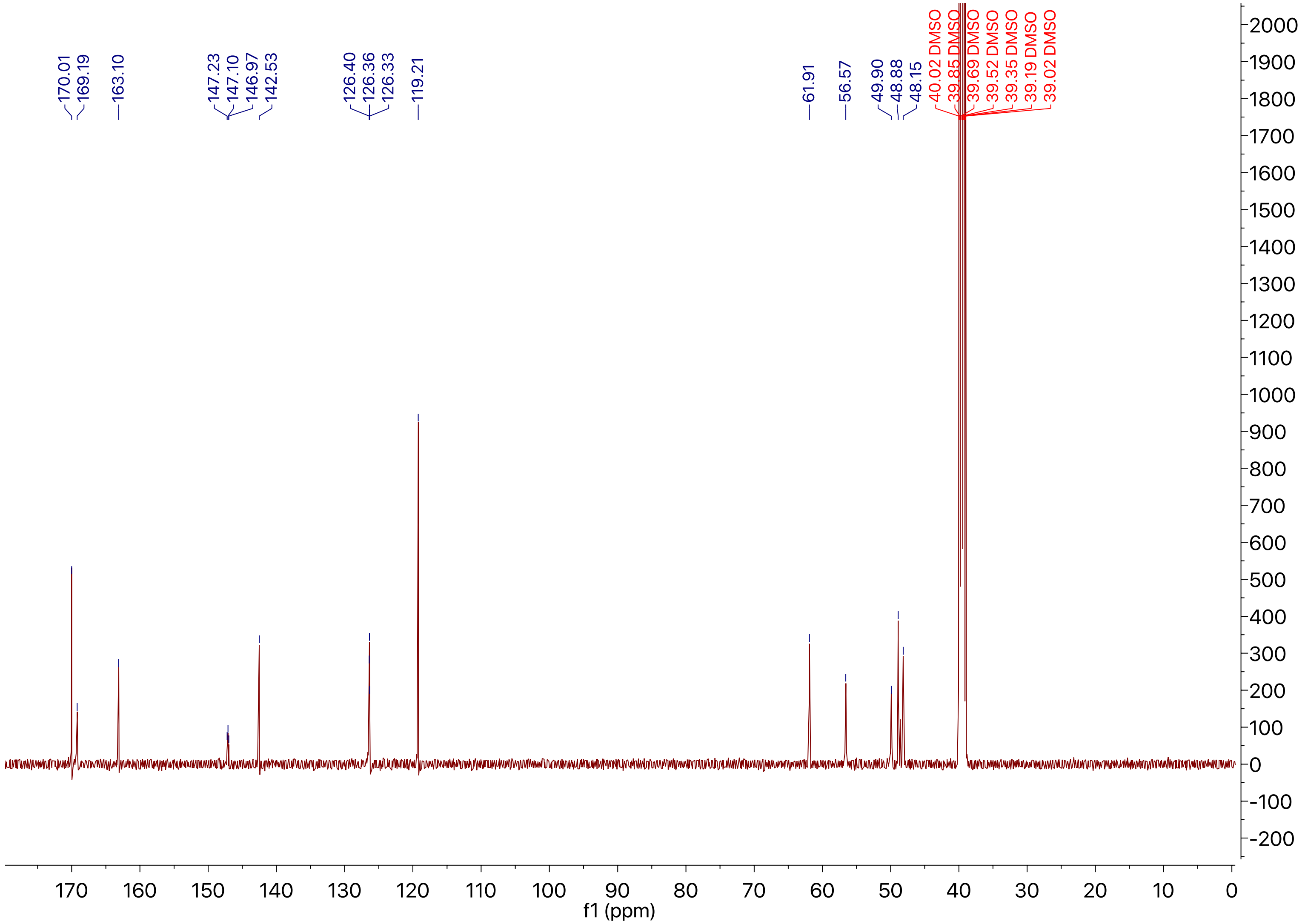

Figure S14. ^{13}C NMR of **NO2ASF$_5$** in d$_6$-DMSO at 25 °C. Sample contains methanol.

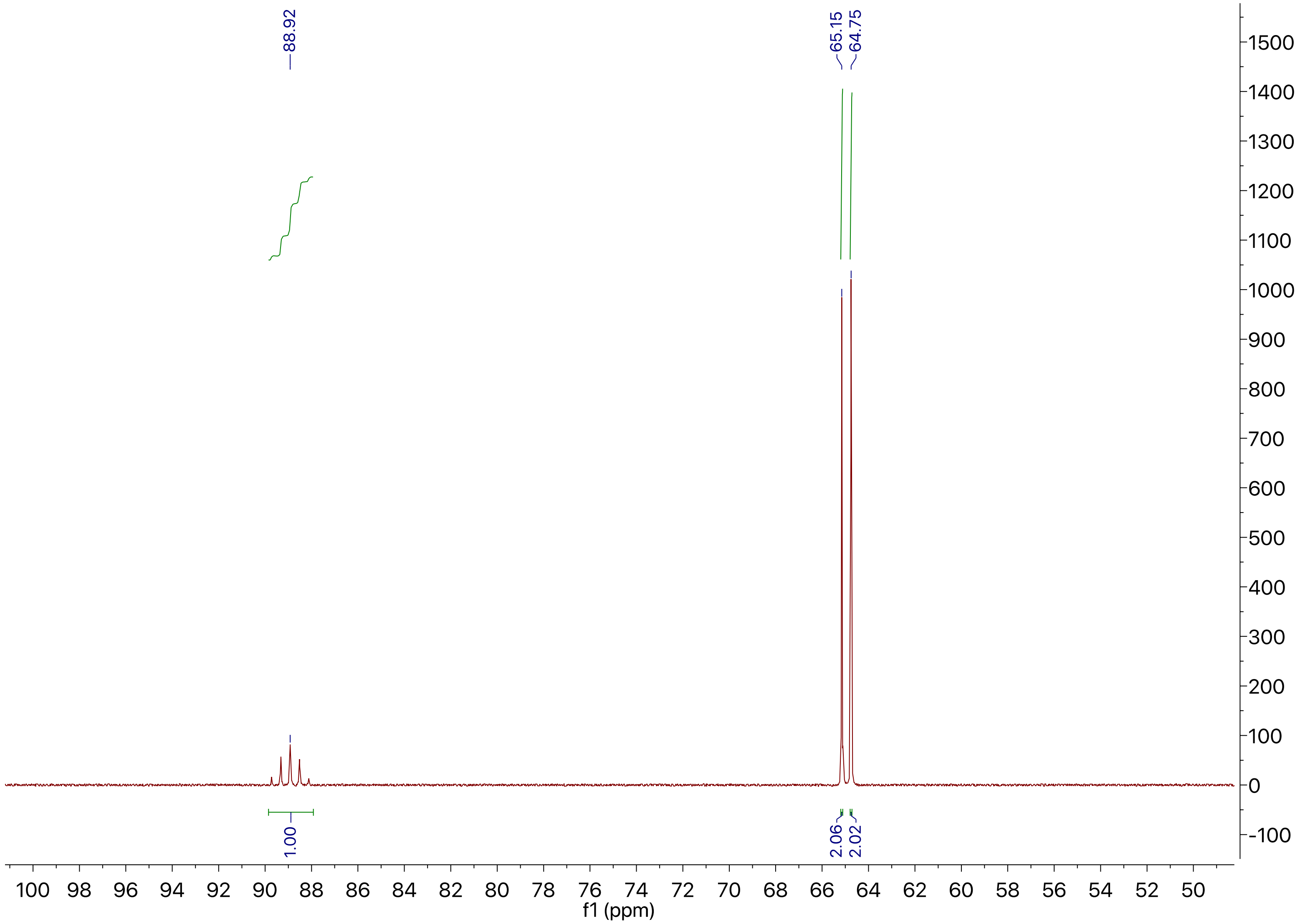

Figure S15. ^{19}F NMR of **NO2ASF$_5$** in d$_6$-DMSO at 25 °C.

Figure S16. ^{19}F NMR of **CoNO2ASF$_5$** in 50mM MES pH 5.5 (red) and CHES pH 9 (blue) buffer in 10% D_2O at 25 °C.
